# Shortening the interval between the first and the second dose of vancomycin facilitates rapid achievement of the target AUC without increasing the risk of acute kidney injury, provided the AUC on the second day is appropriately controlled: a multicenter retrospective study

**DOI:** 10.1186/s40780-025-00452-3

**Published:** 2025-05-26

**Authors:** Tomoyuki Ishigo, Ayako Suzuki, Yuta Ibe, Satoshi Fujii, Masahide Fukudo, Hiroaki Yoshida, Hiroaki Tanaka, Hisato Fujihara, Fumihiro Yamaguchi, Fumiya Ebihara, Takumi Maruyama, Yukihiro Hamada, Yusuke Yagi, Masaru Samura, Fumio Nagumo, Toshiaki Komatsu, Atsushi Tomizawa, Akitoshi Takuma, Hiroaki Chiba, Yoshifumi Nishi, Yuki Enoki, Kazuaki Taguchi, Kazuaki Matsumoto

**Affiliations:** 1 Department of Pharmacy, Sapporo Medical University Hospital, Sapporo, Japan; 2 Laboratory of Clinical Pharmacokinetics, School of Pharmacy, Kitasato University, Sagamihara, Japan; 3 Department of Pharmacy, Kitasato University Hospital, Sagamihara, Japan; 4 Department of Pharmacy, Kyorin University Hospital, Mitaka, Japan; 5 Department of Hospital Pharmaceutics, School of Pharmacy, Showa Medical University, Tokyo, Japan; 6 Department of Respiratory Medicine, Showa Medical University Fujigaoka Hospital, Yokohama, Japan; 7 Department of Pharmacy, Tokyo Women’s Medical University Hospital, Tokyo, Japan; 8 Department of Pharmacy, Kochi Medical School Hospital, Kochi, Japan; 9 Department of Pharmacy, Yokohama General Hospital, Yokohama, Japan; 10 Faculty of Pharmaceutical Sciences, Teikyo Heisei University, Tokyo, Japan; 11 Division of Pharmacodynamics, Keio University Faculty of Pharmacy, Tokyo, 105-8512 Japan; 12 Department of Pharmacy, Showa Medical University Northern Yokohama Hospital, Yokohama, Japan; 13 Department of Pharmacy, Tohoku Kosai Hospital, Sendai, Japan; 14 Center for Pharmacist Education, School of Pharmacy, Nihon University, Funabashi, Japan

**Keywords:** Vancomycin, Therapeutic drug monitoring, Dosing interval, Area under the concentration-time curve, Acute kidney injury

## Abstract

**Background:**

The impact of shortening or extending a vancomycin dosing interval on early attainment of target blood levels and acute kidney injury (AKI) remains unclear. We investigated the relationship between the interval of the first and second doses of vancomycin and early area under the concentration-time curve (AUC) and AKI.

**Methods:**

Patients (≥ 18 years) who started vancomycin and had trough/peak blood samples were included. The definition of shortened interval as the first and second doses of vancomycin was < 12 h. The cumulative incidence of AKI within 21 days was compared using the shortened interval and AUC on day 1 and 2.

**Results:**

Among 668 patients (median age 69 [interquartile range (IQR): 57, 78] years, 40% female), the proportion achieving an AUC ≥ 400 µg·h/mL on day 1 was significantly higher in the shortened-interval group (82% vs. 50%; *p* < 0.001). Multivariate analysis revealed no association between a shortened interval (hazards ratio [HR], 1.10 [95% confidence interval (CI), 0.63–1.91]; *p* = 0.750) or an AUC > 600 µg·h/mL on day 1 alone (HR, 2.17 [95% CI, 0.64–7.42]; *p* = 0.220) and AKI onset. However, an AUC > 600 µg·h/mL on day 2 alone (HR, 2.92 [95% CI, 1.45–5.87]; *p* = 0.003) or on both days (HR, 11.18 [95% CI, 5.07–24.67]; *p* < 0.001) was significantly associated with increased AKI risk.

**Conclusions:**

Shortening the dosing interval facilitates early achievement of target AUC without increasing AKI risk, provided AUC on day 2 is appropriately controlled.

**Supplementary Information:**

The online version contains supplementary material available at 10.1186/s40780-025-00452-3.

## Introduction

Vancomycin is a widely used antibiotic for treating infections caused by methicillin-resistant *Staphylococcus aureus* (MRSA) [1]. The clinical efficacy and potential nephrotoxicity of vancomycin are strongly influenced by the area under the concentration-time curve (AUC) [2, 3]. Notably, early AUCs on days 1 and 2 are closely associated with treatment outcomes, underscoring the importance of optimizing early therapy [4, 5].

To rapidly achieve therapeutic vancomycin concentrations, loading doses are recommended. Current consensus guidelines advocate for a single 25–30 mg/kg loading dose [2], as supported by meta-analyses demonstrating that loading doses increase the likelihood of reaching therapeutic levels early without increasing nephrotoxicity [6, 7]. In contrast, high AUCs, particularly exceeding 500–600 µg·h/mL on the second day, are associated with an increased risk of acute kidney injury (AKI) [5, 8, 9]. Therefore, careful initial dosing and early therapeutic drug monitoring (TDM) are critical.

The second dose is typically administered 12 h after the initial loading dose for patients with normal renal function [2]. However, in clinical practice, the interval between the first and second doses often varies depending on the timing of vancomycin initiation. This variability raises concerns about achieving target AUCs and balancing efficacy with safety.

Therefore, we investigated the shortening dosing the interval between the first and second doses of vancomycin and the achievement of target AUC. In addition, we investigated the relationship between shortening the dosing interval of vancomycin and the AUC on day 1 and 2 and the risk of AKI.

## Patients and methods

### Study participants

**Fig. 1:**
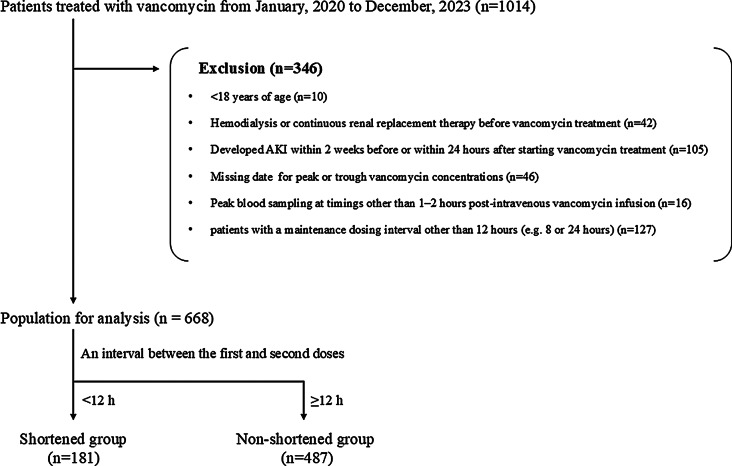
Patient inclusion flowchart for the inclusion of study participants. Abbreviations: AKI, acute kidney injury

This multicenter retrospective observational study was conducted at Sapporo Medical University Hospital, Kyorin University Hospital, Tokyo Women’s Medical University Hospital, Kochi Medical School Hospital, Showa Medical University Fujigaoka Hospital, Yokohama General Hospital, Kitasato University Hospital, Showa Medical University Northern Yokohama Hospital, and Tohoku Kosai Hospital. We retrospectively analyzed data from patients who received vancomycin for at least 24 h and underwent 2-point (trough and peak) blood draws between January 2020 and December 2023. Since guidelines recommend early TDM with 2-point blood collection for patients at high risk of AKI and accurate AUC can be calculated [2]. Blood samples for trough concentrations were collected immediately (within 30 min) before vancomycin administration, and samples for peak concentrations were taken 1–2 h post-intravenous vancomycin infusion (C1-C2) [2]. The enrollment protocol for this study is illustrated in Fig. 1. The exclusion criteria were as follows: patients < 18 years of age, patients on hemodialysis or continuous renal replacement therapy, patients missing data for peak or trough vancomycin concentrations, patients who developed AKI within 2 weeks before or within 24 h after starting vancomycin treatment, patients with peak blood sampling times outside the C1-C2 interval, and patients with a maintenance dosing interval other than 12 h (e.g. 8–24 h). The AUC at TDM was derived from trough and peak levels at the initial TDM using the population parameters of Bayesian estimation software, Practical AUC-guided TDM (PAT) (version 3.0) [10]. Patients were further classified into two groups: those in which the interval between the first dose and the second dose was < 12 h (shortened group) and those in which the interval was ≥ 12 h (non-shortened group). Furthermore, the shortened group was classified into three sub-groups: <6 h, 6–8 h, and > 8 h, and a sub-analysis was performed.

### Data collection

We obtained information on the age, sex, body weight, height, body mass index, laboratory data, and comorbidities, including blood cancer, solid tumors, and heart failure, from the medical records of the patients. The laboratory data comprised serum albumin, serum creatinine (SCr), creatinine clearance (CLcr), serum creatinine-based estimated glomerular filtration rate (eGFRcr), and blood urea nitrogen. In addition, we obtained data on vancomycin dosage, trough and peak vancomycin concentrations, presence of sepsis or septic shock, and concurrent medications such as antimicrobials other than vancomycin, catecholamines, and loop diuretics (including furosemide, azosemide, and torasemide). The internationally validated CKD-EPI formula is not suitable for Japanese, for whom a specific GFR estimation formula exists. Therefore, eGFRcr was calculated based on the Japanese Society of Nephrology formula (Eq. 1) [11]. Furthermore, CLcr was calculated using the Cockcroft-Gault (CG) formula, considering that the Japanese vancomycin and PAT guidelines use CLcr as the dosing criterion (Eq. 2) [12].

1 $$\begin{aligned}&\:\text{e}\text{G}\text{F}\text{R}\text{c}\text{r}\:(\text{m}\text{L}/\text{m}\text{i}\text{n}/1.73\:{\text{m}}^{2})\\&\quad=194\:\times\:{\text{S}\text{C}\text{r}}^{-1.094}\times\:{\text{A}\text{g}\text{e}}^{-0.287}\:\left(\times\:0.739\:\text{i}\text{f}\:\text{f}\text{e}\text{m}\text{a}\text{l}\text{e}\right)\end{aligned}$$

2 $$\begin{aligned}\:\text{C}\text{L}\text{c}\text{r}\:(\text{m}\text{L}/\text{m}\text{i}\text{n})&=\:\frac{\left(140-\text{A}\text{g}\text{e}\right)\:\times\:\:\text{B}\text{o}\text{d}\text{y}\:\text{W}\text{e}\text{i}\text{g}\text{h}\text{t}\:\left(\text{k}\text{g}\right)}{72\:\times\:\:\text{S}\text{C}\text{r}\:\left(\text{m}\text{g}/\text{d}\text{L}\right)}\:\\&\quad\left(\times\:0.85\:\text{i}\text{f}\:\text{f}\text{e}\text{m}\text{a}\text{l}\text{e}\right)\end{aligned}$$

### Outcomes and definition

The efficacy outcome was the rate of achieving a day 1 AUC (AUC_0 − 24 h_) of 400 µg·h/mL or greater [4, 5]. The safety outcomes were evaluated based on the AUC on days 1 and 2 and the cumulative incidence of AKI. The risk of AKI was assessed by examining the percentage of patients with an AUC_0 − 24 h_ and AUC_24 − 48 h_ >600 µg·h/mL, AUC_0 − 24 h_ and AUC_24 − 48 h_ >500 µg·h/mL [5, 8, 9, 13]. The cumulative incidence of AKI was compared between the shortened and non-shortened groups, and the AUC on days 1 and 2 was also compared between the four groups. The four groups based on AUC were classified as follows: group with AUC of ≤ 600 µg·h/mL on both day 1 and day 2, group with AUC of > 600 µg·h/mL on day 1 alone, group with AUC of > 600 µg·h/mL on day 2 alone, or group with AUC of > 600 µg·h/mL on both days. In addition, the cutoff value was examined at 500 µg·h/mL. The cumulative incidence of AKI was compared between patients in the intensive care unit (ICU) and non-ICU. AKI was evaluated during the vancomycin treatment period or until day 21. In addition, we examined early AKI within 7 days, which was probably most affected by the shortened doses and early AUCs in this study. Shortening the dosing interval was defined as an interval of < 12 h between the first and second doses. AKI was defined according to the Kidney Disease Improving Global Outcomes criteria (i.e., an increase in SCr of ≥ 0.3 mg/dL [within 48 h], of ≥ 50% from the most recent pre-treatment data during therapy, or continuation of a urine volume of 0.5 mL/kg/h for ≥ 6 h) [14]. Sepsis and septic shock were defined according to the Sepsis-3 criteria [15].

### Statistical analyses

Data were presented as median (interquartile range [IQR]) or frequency (percentage). Comparisons between both groups were conducted using the Mann–Whitney U test for continuous variables and the chi-square test or Fisher’s exact test for nominal variables. Receiver operating characteristic curves were drawn to calculate the area under the curve, and the optimal cutoff values of the predicting factor for AUC > 600 or > 500 µg·h/mL on day 1 at the initial TDM. The Youden Index was used to determine the optimal cut-off value. The Gray test and Fine-Gray model were used to assess the cumulative frequency of AKI while adjusting for mortality as a competing risk. Factors with a p-value < 0.1 in univariate analysis were included in the multivariate analysis to identify the factors associated with AKI. Statistical significance was set at a p-value < 0.05. The Bonferroni correction was used to compare the four groups to account for the increased alpha error. A p-value < 0.0167 was statistically significant to perform statistical analysis three times with the control group. Statistical analyses were performed using JMP Pro 17.0 (SAS Institute Inc., Cary, NC, USA) and EZR version 1.54 (Saitama Medical Center, Jichi Medical University, Saitama, Japan), which is a R graphical user interface (The R Foundation for Statistical Computing, Vienna, Austria).

## Results

### Baseline clinical characteristics

A total of 668 patients were included in this study after applying the exclusion criteria (Fig. 1). The median age of the patients was 69 years (IQR: 57, 78), and 40% were female. A total of 181 and 487 patients were in the shortened and non-shortened groups, respectively (Fig. 1 and Fig. S1). Most of the non-shortened group had maintenance dosing initiated 12 h after the first dose, but the interval between the first and second dose in the shortened group was generally variable (Fig. S1).

### Comparison of patient backgrounds between the shortened and non-shortened groups

There were no significant differences in age or sex between the shortened and non-shortened groups (Table 1). The proportion of patients in the ICU was significantly higher in the shortened group than in the non-shortened group (43% [77/181] vs. 30% [147/487], *p* = 0.003) (Table 1). In contrast, the sepsis and septic shock proportions were not significantly different between both groups (Table 1). There were no significant differences between the shortened and non-shortened groups regarding SCr, CLcr, or the percentage of concomitant medications used. The initial dose was significantly higher in the shortened group compared to the non-shortened group (27.1 mg/kg [IQR: 23.1, 30.0] vs. 26.1 mg/kg [IQR: 23.1, 30.0], *p* = 0.005); however, the percentage of loading doses ≥ 25 mg/kg was not significantly different between both groups (68% [123/181] vs. 62% [300/487], *p* = 0.130) (Table 1). There was no significant difference between the two groups in the total dose of vancomycin administered during the first and second doses (41.1 mg/kg [36.3, 46.0] vs. 40.8 mg/kg [35.5, 46.2], *p* = 0.322) (Table 1).

**Table 1:** Characteristics of the patients

	Non-shortened group (*n* = 487)	Shortened group (*n* = 181)	*p*-value
Age, years	69 (58, 77)	70 (54, 79)	0.776
Female, n (%)	201 (41%)	63 (35%)	0.129
Height, cm	162 (155, 170)	163 (155, 170)	0.576
Body weight, kg	56 (49, 66)	56 (48, 67)	0.981
BMI, kg/m^2^	21.6 (19.0, 24.7)	21.3 (18.6, 24.6)	0.456
ICU, n (%)	147 (30%)	77 (43%)	0.003
Sepsis, n (%)	104 (21%)	38 (21%)	0.919
Septic shock, n (%)	44 (9%)	9 (5%)	0.084
Bacteremia, n (%)	200 (41%)	89 (49%)	0.063
Comorbidities, n (%)			
Blood cancer	46 (9%)	10 (6%)	0.104
Solid tumor	157 (32%)	43 (24%)	0.033
Heart failure	105 (22%)	51 (28%)	0.072
Diabetes mellitus	143 (29%)	50 (28%)	0.659
Laboratory data			
SCr, mg/dL	0.68 (0.53, 0.87)	0.68 (0.55, 0.87)	0.988
CLcr, mL/min	77.3 (57.5, 104.0)	77.3 (54.5, 102.1)	0.751
eGFRcr, mL/min/1.73 m^2^	78.4 (63.4, 100.6)	79.6 (62.0, 103.6)	0.726
BUN, mg/dL	16 (12, 22)	16 (12, 23)	0.900
BUN/SCr	23.3 (16.8, 32.6)	23.6 (17.2, 32.9)	0.871
Concomitant medication, n (%)			
Meropenem	190 (39%)	66 (36%)	0.547
Tazobactam/piperacillin	91 (19%)	27 (15%)	0.256
Cefepime	28 (6%)	18 (10%)	0.057
Loop diuretics	112 (23%)	32 (18%)	0.137
Catecholamine	87 (18%)	36 (20%)	0.548
Aminoglycoside	18 (4%)	8 (4%)	0.667
Vancomycin therapy (up to initial TDM)			
Initial dose, mg/kg	26.1 (22.8, 28.8)	27.1 (23.1, 30.0)	0.005
Loading dose (≥ 25 mg/kg), n (%)	300 (62%)	123 (68%)	0.130
Combined amount of the first and second doses, mg/kg	40.8 (35.5, 46.2)	41.1 (36.3, 46.0)	0.322
Maintenance dose, mg/kg/day	30.0 (23.7, 36.4)	28.8 (22.1, 35.9)	0.147
Number of doses before initial TDM	3 (2, 3)	2 (2, 4)	0.586
AUC at initial TDM			
AUC_0 − 24 h_, µg·h/mL	401 (335, 457)	495 (426, 558)	< 0.001
AUC_24 − 48 h_, µg·h/mL	430 (353, 507)	444 (370, 522)	0.091
AUC_ss_, µg·h/mL	461 (372, 567)	440 (362, 545)	0.200

The data are presented as median (interquartile range) or number (percentage). Statistical significance was set at *p* < 0.05. Abbreviations: BMI, body mass index; ICU, intensive care unit; CLcr, creatinine clearance; eGFRcr, creatinine-based estimated glomerular filtration rate; BUN, blood urea nitrogen; BUN/SCr, ratio of blood urea nitrogen to serum creatinine; TDM, therapeutic drug monitoring; AUC, area under the concentration-time curve. AUC_0 − 24 h_, AUC on day 1; AUC_24 − 48 h_, AUC on day 2; AUC_ss_, AUC at steady state

### AUC on day 1 and day 2 in the shortened and non-shortened groups

The proportion of patients with AUC_0 − 24 h_ ≥400 µg·h/mL (82% [149/181] vs. 50% [245/487], *p* < 0.001) or AUC_0 − 24 h_ >600 µg·h/mL (14% [26/181] vs. 1% [6/487], *p* < 0.001) were significantly higher in the shortened group compared with the non-shortened group (Table 2). Similarly, the proportion of patients with AUC_0 − 24 h_ ≥400 µg·h/mL (87% [107/123] vs. 61% [183/300], *p* < 0.001) or AUC_0 − 24 h_ >600 µg·h/mL (16% [20/123] vs. 1% [4/300] when examining patients who received a loading dose of ≥ 25 mg/kg, *p* < 0.001) were significantly higher in the shortened group than the non-shortened (Table 2). The proportion of patients with AUC_0 − 24 h_ ≥400 µg·h/mL (84% [88/105] vs. 56% [149/265], *p* < 0.001) or AUC_0 − 24 h_ >600 µg·h/mL (16% [17/105] vs. 2% [4/265], *p* < 0.001) were significantly higher in the shortened group compared to the non-shortened group when examining patients who received a combined amount of the first and second doses of ≥ 40 mg/kg (Table 2). A similar trend was observed in patients who received a loading dose of < 25 mg/kg or a combined total of < 40 mg/kg for the first and second doses (Table 2). The proportion of patients with AUC_0 − 24 h_ >600 µg·h/mL was significantly higher in the shortened group than in the non-shortened group (Table 2), but the percentage of patients with AUC_24 − 48 h_ >600 µg·h/mL was not different between the two groups (Table 2). The same results were obtained when the cutoff value for AUC was set at 500 µg·h/mL (Table 2). A similar study was performed on patients in the ICU, and a similar trend was observed (Table S1).

**Table 2:** Comparison of the proportion of patients with AUCs in the shortened and non-shortened groups

**All (*n*** **= 668)**	**Non-shortened group** **(*n*** **= 487)**	**Shortened group** **(*n*** **= 181)**	***p*** **-value**
AUC_0 − 24 h_ ≥400 µg·h/mL	245 (50%)	149 (82%)	< 0.001
AUC_0 − 24 h_ >500 µg·h/mL	64 (13%)	87 (48%)	< 0.001
AUC_0 − 24 h_ >600 µg·h/mL	6 (1%)	26 (14%)	< 0.001
AUC_24 − 48 h_ ≥400 µg·h/mL	295 (61%)	121 (67%)	0.137
AUC_24 − 48 h_ >500 µg·h/mL	130 (27%)	57 (31%)	0.702
AUC_24 − 48 h_ >600 µg·h/mL	45 (9%)	15 (8%)	0.702
**Initial dose; <25 mg/kg (*n*** **= 245)**	**Non-shortened group** **(*n*** **= 187)**	**Shortened group** **(*n*** **= 58)**	
AUC_0 − 24 h_ ≥400 µg·h/mL	62 (33%)	42 (72%)	< 0.001
AUC_0 − 24 h_ >500 µg·h/mL	19 (10%)	18 (31%)	< 0.001
AUC_0 − 24 h_ >600 µg·h/mL	2 (1%)	6 (10%)	< 0.001
AUC_24 − 48 h_ ≥400 µg·h/mL	106 (57%)	34 (59%)	0.795
AUC_24 − 48 h_ >500 µg·h/mL	42 (22%)	14 (24%)	0.790
AUC_24 − 48 h_ >600 µg·h/mL	15 (8%)	3 (5%)	0.576
**Initial dose; ≥25 mg/kg (*n*** **= 423)**	**Non-shortened group** **(*n*** **= 300)**	**Shortened group** **(*n*** **= 123)**	
AUC_0 − 24 h_ ≥400 µg·h/mL	183 (61%)	107 (87%)	< 0.001
AUC_0 − 24 h_ >500 µg·h/mL	45 (15%)	69 (56%)	< 0.001
AUC_0 − 24 h_ >600 µg·h/mL	4 (1%)	20 (16%)	< 0.001
AUC_24 − 48 h_ ≥400 µg·h/mL	189 (63%)	87 (71%)	0.129
AUC_24 − 48 h_ >500 µg·h/mL	88 (29%)	43 (35%)	0.939
AUC_24 − 48 h_ >600 µg·h/mL	30 (10%)	12 (10%)	0.939
**Combined amount of the first and second doses; <40 mg/kg (*n*** **= 298)**	**Non-shortened group** **(*n*** **= 222)**	**Shortened group** **(*n*** **= 76)**	
AUC_0 − 24 h_ ≥400 µg·h/mL	96 (43%)	61 (80%)	< 0.001
AUC_0 − 24 h_ >500 µg·h/mL	31 (14%)	32 (42%)	< 0.001
AUC_0 − 24 h_ >600 µg·h/mL	2 (1%)	9 (12%)	< 0.001
AUC_24 − 48 h_ ≥400 µg·h/mL	119 (54%)	49 (64%)	0.099
AUC_24 − 48 h_ >500 µg·h/mL	46 (21%)	19 (25%)	0.436
AUC_24 − 48 h_ >600 µg·h/mL	11 (5%)	2 (3%)	0.527
**Combined amount of the first and second doses; ≥40 mg/kg (*n*** **= 370)**	**Non-shortened group** **(*n*** **= 265)**	**Shortened group** **(*n*** **= 105)**	
AUC_0 − 24 h_ ≥400 µg·h/mL	149 (56%)	88 (84%)	< 0.001
AUC_0 − 24 h_ >500 µg·h/mL	33 (12%)	55 (52%)	< 0.001
AUC_0 − 24 h_ >600 µg·h/mL	4 (2%)	17 (16%)	< 0.001
AUC_24 − 48 h_ ≥400 µg·h/mL	176 (66%)	72 (69%)	0.691
AUC_24 − 48 h_ >500 µg·h/mL	84 (32%)	38 (36%)	0.407
AUC_24 − 48 h_ >600 µg·h/mL	34 (13%)	13 (12%)	0.907

The data are presented as number (percentage). Statistical significance was set at *p* < 0.05. Abbreviations: AUC, area under the concentration-time curve. AUC_0 − 24 h_, AUC on day 1; AUC_24 − 48 h_, AUC on day 2

### Relationship between time to a second dose of vancomycin, loading dose or the combined amount of the first and second doses, and AUC

No significant association between the interval from the first to second dose and the loading dose or combined amount of the first and second doses was observed (Figs. S2 and S3). There was a significant negative correlation between the interval from first to second dose, AUC_0 − 24 h_ (*r* = − 0.4246, *p* < 0.001), and AUC_24 − 48 h_ (*r* = − 0.1335, *p* < 0.001). No significant correlation with AUC on the steady state was observed (*r* = 0.0550, *p* = 0.156) (Fig. S4).

### Comparison of differences in the shortening time of the shortened group, VCM dosage, and AUC

The shortened groups were further classified into three sub-groups using the difference in shortening time: <6 h, 6–8 h, and > 8 h (*n* = 12, 76, and 93, respectively), and the dosage and AUC were compared (Table 3). There was no significant difference in the initial dose or combined amount of the first and second doses among the three groups. There was a significant difference in AUC_0 − 24 h_ at the time of initial dose design among the three groups (*p* < 0.001) (Table 3). Specifically, the group with > 8 h had a higher percentage of AUC_0 − 24 h_ at an initial TDM of < 400 µg·h/mL, whereas the group with < 6 h had a higher percentage of AUC_0 − 24 h_ at an initial TDM of > 600 µg·h/mL. Regarding the 6–8 h group, 21% (16/76) had AUC_0 − 24 h_ at an initial TDM of > 600 µg·h/mL. Therefore, factors associated with an AUC_0 − 24 h_ of > 600 µg·h/mL and the cutoff values were examined. Furthermore, it was demonstrated that the initial and total dose of the first and second administrations did not predict AUC_0 − 24 h_ accurately at an initial TDM of > 600 µg·h/mL (Fig. S5). In contrast, we demonstrated that the AUC_0 − 24 h_ at the initial dose design predicted the AUC_0 − 24 h_ accurately at an initial TDM of > 600 µg·h/mL and with an optimal cutoff value of 583 µg·h/mL (Fig. S5). Furthermore, the optimal cutoff value for predicting values of AUC_0 − 24 h_ at an initial TDM of > 500 µg·h/mL was 538 µg·h/mL (Fig. S6).

**Table 3:** Subanalysis by shortening time in the shortened group

All shortened patients (*n* = 181)	Interval between the first dose and the second dose			*p*-value
	< 6 h (*n* = 12)	6–8 h (*n* = 76)	> 8 h (*n* = 93)	
Initial dose, mg/kg	24.0 (22.3, 30.9)	26.8 (23.8, 30.0)	27.3 (23.0, 30.6)	0.673
Loading dose (≥ 25 mg/kg), n (%)	5 (42%)	54 (71%)	64 (69%)	0.124
Combined amount of the first and second doses, mg/kg	36.4 (33.4, 45.6)	41.6 (35.4, 46.9)	41.1 (36.8, 45.9)	0.560
Maintenance dose, mg/kg/day	26.5 (22.0, 32.5)	30.0 (20.7, 36.7)	28.7 (22.2, 35.6)	0.795
AUC_0 − 24 h_ at the time of initial dose design	561 (509, 598)	505 (455, 587)	468 (402, 521)	< 0.001
AUC_0 − 24 h_ at initial TDM				0.007
> 600 µg·h/mL	3 (25%)	16 (21%)	7 (8%)	
400–600 µg·h/mL	7 (58%)	54 (71%)	62 (67%)	
< 400 µg·h/mL	2 (17%)	6 (8%)	24 (26%)	

The data are presented as median (interquartile range) or number (percentage). Statistical significance was set at *p* < 0.05. Abbreviations: TDM, therapeutic drug monitoring; AUC, area under the concentration-time curve. AUC_0 − 24 h_, AUC on day 1

### Incidence of AKI and its risk factor

**Fig. 2:**
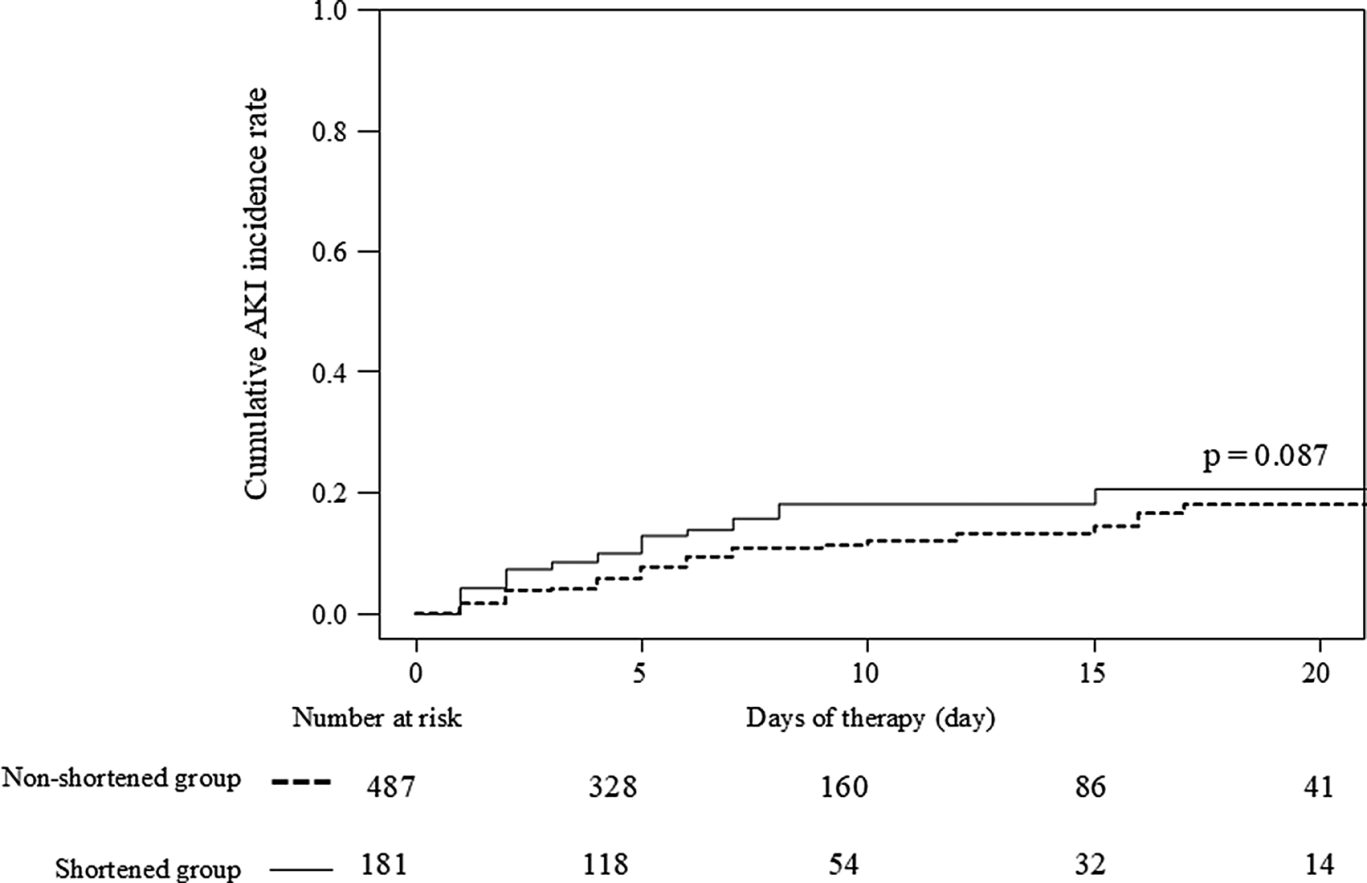
Relationship between the presence or absence of shortened dosing intervals and the cumulative rate of AKI within 21 days. Abbreviations: AKI, acute kidney injury

The overall incidence of AKI within 21 days was 12% (77/668). There was no significant difference in the cumulative incidence of AKI between the shortened and non-shortened groups (*p* = 0.087) (Fig. 2). There was a significant difference in the cumulative incidence of AKI between the four groups with different early AUCs (the AUC_0 − 24 h_ [≤ 600 µg·h/mL or > 600 µg·h/mL, respectively,] and AUC_24 − 48 h_ [≤ 600 µg·h/mL or > 600 µg·h/mL]) (Fig. S7a). The same results were obtained when the cutoff value for AUC was set at 500 µg·h/mL (Fig. S7b). Patients in the ICU had a significantly higher cumulative AKI incidence rate than patients who were non-ICU (Fig. S8). Conversely, no statistically significant difference was observed in cumulative AKI rates between shortened and non-shortened doses in patients in the ICU (Fig. S9).

### Association between risk factors and the probability of developing AKI

In univariate analyses, patients in the ICU (hazard ratio [HR], 2.29 [95% confidence interval (CI), 1.47–3.57]; *p* < 0.001), use of tazobactam/piperacillin (HR, 3.06 [95%CI, 1.94–4.81]; *p* < 0.001), use of catecholamine (HR, 2.73 [95%CI, 1.71–4.37]; *p* < 0.001), and use of loop diuretic (HR, 1.92 [95%CI, 1.21–3.04]; *p* = 0.005) were significantly associated with AKI onset (Table 4). Using the group with AUC ≤ 600 µg·h/mL on both days 1 and 2 as the reference, the risk of AKI was not significantly higher in the group with an AUC > 600 µg·h/mL on day 1 alone, but the group with an AUC > 600 µg·h/mL on day 2 alone or on both days had a significantly higher risk of AKI onset (Table 4).

**Table 4:** Fine-Gray analyses of factors associated with AKI within 21 days

	Univariate model			Multivariate model 1			Multivariate model 2		
	HR	(95% CI)	*p*-value	HR	(95% CI)	*p*-value	HR	(95% CI)	*p*-value
Age, per 1-year increase	1.00	0.99–1.02	0.830						
Sex; female	0.89	0.57–1.39	0.600						
BMI, per 1 kg/m^2^ increase	1.02	0.99–1.06	0.230						
ICU	2.29	1.47–3.57	< 0.001	1.39	0.77–2.50	0.280	1.56	0.87–2.78	0.140
Sepsis	1.25	0.75–2.08	0.400						
Septic shock	1.95	0.98–3.86	0.057	1.05	0.46–2.39	0.900	1.11	0.48–2.58	0.800
Vancomycin therapy (up to initial TDM)									
Shortened interval	1.50	0.94–2.39	0.088	1.10	0.63–1.91	0.750	1.13	0.65–1.97	0.660
Loding dose, ≥ 25 mg/kg	1.23	0.77–1.99	0.390						
Vancomycin AUC									
AUC_0 − 24 h_ ≤600 µg·h/mL and AUC_24 − 48 h_ ≤600 µg·h/mL	Reference	-	-	Reference	-	-			
AUC_0 − 24 h_ >600 µg·h/mL and AUC_24 − 48 h_ ≤600 µg·h/mL	2.28	0.76–6.88	0.140	2.17	0.64–7.42	0.220			
AUC_0 − 24 h_ ≤600 µg·h/mL and AUC_24 − 48 h_ >600 µg·h/mL	2.70	1.34–5.44	0.006	2.92	1.45–5.87	0.003			
AUC_0 − 24 h_ >600 µg·h/mL and AUC_24 − 48 h_ >600 µg·h/mL	10.26	5.00-21.04	0.006	11.18	5.07–24.67	< 0.001			
AUC_0 − 24 h_ ≤500 µg·h/mL and AUC_24 − 48 h_ ≤500 µg·h/mL	Reference	-	-				Reference	-	-
AUC_0 − 24 h_ >500 µg·h/mL and AUC_24 − 48 h_ ≤500 µg·h/mL	1.62	0.57–4.58	0.360				1.68	0.55–5.16	0.360
AUC_0 − 24 h_ ≤500 µg·h/mL and AUC_24 − 48 h_ >500 µg·h/mL	2.10	1.05–4.22	0.037				2.53	1.20–5.32	0.015
AUC_0 − 24 h_ >500 µg·h/mL and AUC_24 − 48 h_ >500 µg·h/mL	5.10	3.10–8.45	< 0.001				6.01	3.40-10.61	< 0.001
Tazobactam/piperacillin	3.06	1.94–4.81	< 0.001	3.69	2.31–5.88	< 0.001	4.36	2.67–7.10	< 0.001
Catecholamine	2.73	1.71–4.37	< 0.001	1.81	0.91–3.61	0.089	1.74	0.91–3.31	0.095
Loop diuretics	1.92	1.21–3.04	0.005	1.29	0.73–2.27	0.380	1.00	0.56–1.79	1.000

Statistical significance was set at *p* < 0.05. To account for the increased alpha error, the Bonferroni correction was used to compare the four groups. To perform statistical analysis three times versus the control group, a p-value < 0.0167 was considered statistically significant. Abbreviations: AKI, acute kidney injury; HR, hazard ratio; CI, confidence interval; BMI, body mass index; ICU, intensive care unit; AUC, area under the concentration-time curve; AUC_24 − 48 h_, AUC on day 2

In multivariate analysis, shortened interval was not associated with AKI onset (HR, 1.10 [95% CI, 0.63–1.91]; *p* = 0.750). In contrast, use of tazobactam/piperacillin was significantly associated with AKI onset (HR, 3.69 [95% CI, 2.31–5.88]; *p* < 0.001). In addition, the risk of AKI was not significantly higher in the group with an AUC > 600 µg·h/mL on day 1 (HR, 2.17 [95% CI, 0.64–7.42]; *p* = 0.220), but the group with an AUC > 600 µg·h/mL on day 2 alone (HR, 2.92 [95%CI, 1.45–5.87]; *p* = 0.003) and the group with an AUC > 600 µg·h/mL on both days (HR, 11.18 [95%CI, 5.07–24.67]; *p* < 0.001) had a significantly higher risk of AKI onset (Table 4, model 1). The same results were obtained when the cutoff value for AUC was set at 500 µg·h/mL (Table 4, model 2).

A similar study was conducted for early AKI within 7 days, and the shortened dose was not a significant factor in early AKI (Table S2). In addition, the risk of early AKI was not significantly higher in the group with an AUC > 600 µg·h/mL on day 1 alone, but the group with an AUC > 600 µg·h/mL on day 2 and the group with an AUC > 600 µg·h/mL on both days had a significantly higher risk of early AKI onset (Table S2, model A). The same results were obtained when the cutoff value for AUC was set at 500 µg·h/mL (Table. S2, model B).

## Discussion

The results of this study revealed that shortening the interval between the first and second doses of vancomycin is an attractive strategy for achieving the target AUC more quickly without increasing the incidence of AKI, as long as the AUC on the second day is controlled appropriately to be ≤ 600 µg·h/mL (or ≤ 500 µg·h/mL).

Target vancomycin concentrations must be achieved early in the treatment process to improve clinical outcomes. Furthermore, ≥ 400 µg·h/mL on day 1 is associated with early efficacy [4, 5], and loading doses increase the probability of an ≥ 400 µg·h/mL on day 1 [16, 17] and loading doses are associated with early efficacy [4–7]. Casapao et al. [18] reported that in patients with MRSA bacteremia, higher day 1 vancomycin exposure was associated with lower clinical failure and sustained bacteremia rates. Therefore, the initial loading dose is important, as recommended in the guidelines [2]. However, it has been reported that the target vancomycin blood level is not reached in half of the cases even after a loading dose [19]. Therefore, the administration strategy after the loading dose was also considered important, but the method of administration has not been fully clarified. This study revealed that shortening the dosing interval, with or without the loading dose, increases the probability of achieving the target AUC. Furthermore, AUC ≥ 400 µg·h/mL could be achieved from day 1 in approximately 90% of cases if the first loading dose (≥ 25 mg/kg) was combined with a shortened dosing interval (Table 2). In contrast, only 60% of the patients in the non-shortened group had an AUC ≥ 400 µg·h/mL on the first day despite a loading dose of ≥ 25 mg/kg (Table 2). Therefore, it is clarified that in addition to the loading dose, paying attention to the dosing interval when designing the dosing in terms of efficacy is important.

This shortened dosing strategy should be considered as a separate strategy from the determination of loading or daily dose, and it is not conducted by dividing the loading dose. Specifically, if the total dose on the first and second days was the same, shortening the dosing interval may improve the proportion of AUC ≥ 400 µg·h/mL on the first day by approximately 30–40% (Table 2). On the other hand, there is a high possibility of the AUC > 600 µg·h/mL (Table 2). Therefore, determining the initial dose and dosing interval while considering both efficacy and safety is crucial. Regarding the extent of the dosing interval, 6–8 h was appropriate when considering efficacy and safety. Furthermore, when shortening the dosing interval to 6–8 h, confirming the AUC at the initial dosing design and not just the dose, further enhances safety. Specifically, to ensure that AUC on the first day does not > 600 µg·h/mL, the predicted AUC at the time of dose design should be set at < 583 µg·h/mL (Fig. S5). Similarly, to ensure that AUC on the first day is not > 500 µg·h/mL, the predicted AUC should be set at < 538 µg·h/mL (Fig. S6). Accordingly, it was demonstrated that adjusting the initial dose is necessary to align with these targets.

Shortened dosing intervals may have many advantages regarding efficacy; however, there are concerns about side effects. Therefore, we investigated the association between shortened dosing intervals and AUC on days 1 and 2 and AKI, suggesting that shortened dosing intervals may not increase the risk of AKI if the AUC on the second day is appropriately controlled to ≤ 600 µg·h/mL (≤ 500 µg·h/mL). Previous reports have shown that the first loading dose does not increase the risk of AKI [6, 7], but an AUC > 600 µg·h/mL^5^ or > 515 µg·h/mL^8^ on day 2 increases the risk of AKI. Zasowski et al. reported that nephrotoxicity from vancomycin was significantly higher in patients with AUC_0 − 24 h_ >677 µg·h/mL, and AUC_24 − 48 h_ >683 µg·h/mL [13]. Endo et al. reported that AUC cutoff values of > 470.8 µg·h/mL for AUC_0 − 24 h_, > 473.0 µg·h/mL for AUC_24 − 48 h_ were associated with early AKI [20]. Although some previous reports have reported that AUC on day 1 is associated with AKI [13, 20], the association with AUC on day 2 and beyond in those cases has not been evaluated, and the possibility that the results reflect the accumulation of AUCs on day 2 and overall cannot be excluded. In this study, there were significantly more cases with an AUC > 600 µg·h/mL on day 1 in the shortened group; nevertheless, the percentage of patients with an AUC > 600 µg·h/mL on day 2 was not significantly different between the shortened and non-shortened groups. This indicated that the frequency of AKI was not different in the two groups. Furthermore, the risk of developing AKI was not significantly higher in the group with an AUC > 600 µg·h/mL (or > 500 µg·h/mL) on day 1 alone, the risk of developing AKI was significantly higher in the group with an AUC > 600 µg·h/mL (or > 500 µg·h/mL) on day 2 alone and on both days. This supports the hypothesis mentioned earlier. In other words, this result emphasizes the importance of appropriate dosing through early TDM, as indicated in the guidelines [2]. This is due to the risk of AKI being more closely related to the AUC on day 2 and thereafter, rather than on the first day. In addition, regarding the target AUC on day 2, there is a controversy over whether it should be set at ≤ 600 or ≤ 500 µg·h/mL. However, recent reports have suggested safety-related cutoff values of 515 µg·h/mL^8^ or 473.0 µg·h/mL^20^. Furthermore, based on our previous findings that an AUC of 500–600 µg·h/mL on day 2 is a risk of AKI^9^, we considered it desirable to design the initial dosing regimen to ensure that the AUC remains ≤ 500 µg·h/mL to ensure safety.

The combination of tazobactam/piperacillin and vancomycin has been reported to carry a higher risk of AKI than the combination of tazobactam/piperacillin and other β-lactams [21–23]. In this study, we demonstrated that the combination of tazobactam/piperacillin and vancomycin is an independent risk factor for AKI regardless of AUCs (Table 2 and Table S1), supporting previous reports. Presumably, concomitant use of tazobactam/piperacillin is an independent risk factor in relation to serum concentrations of vancomycin [21–24]. In addition, additive nephrotoxic effects of acute interstitial nephritis and direct cell necrosis have been reported in several studies [25, 26]. Alternatively, some recent reports have described an apparent increase in SCr [27]. Therefore, when vancomycin and tazobactam/piperacillin are combined, closer renal function monitoring and strict adjustment of the vancomycin dose should be considered.

The present study has several limitations. First, due to its retrospective observational nature and targeting two-point blood collections after vancomycin initiation, some selection bias may have occurred within the study population. However, as patient data were extracted from nine different Japanese hospitals, the selection bias was possibly smaller than that of a single-hospital study. Second, interpreting these results is difficult as it is unclear whether an elevated AUC increases the risk of AKI or whether AKI increases the likelihood of an elevated AUC [22]. However, our results suggest that the group with an AUC > 600 µg·h/mL on day 1 only and on day 2 only and on both days were designed with higher doses than those associated with the low AUC group from the initial dosing (Table S3). This may explain why an elevated AUC increases the risk of AKI. Finally, we did not investigate the usefulness of shortening the interval between the first and second doses of vancomycin, which should be addressed in future research.

This study revealed that shortening the interval between the first and second doses of vancomycin facilitates early achievement of the target AUC without increasing AKI, provided AUC on day 2 is appropriately controlled. These results emphasize the importance of early TDM and may help in the optimal treatment regimen of vancomycin in clinical practice.

## Electronic supplementary material

Below is the link to the electronic supplementary material.

Supplementary Material 1

Supplementary Material 2

## Data Availability

The data that support the findings of this study are available upon reasonable request.
