# Supplementary material for: Shortening the interval between the first and the second dose of vancomycin facilitates rapid achievement of the target AUC without increasing the risk of acute kidney injury, provided the AUC on the second day is appropriately controlled: a multicenter retrospective study
Source: J Pharm Health Care Sci. 2025 May 26;11:44. doi: 10.1186/s40780-025-00452-3 (PMC12105161; doi:10.1186/s40780-025-00452-3)
Supplement: Supplementary file 1 — Supplementary Material 1 [file 40780_2025_452_MOESM1_ESM.docx]

**Table S1** Comparison of the proportion of patients with AUCs in the shortened and non-shortened groups in critically ill patients

| All (n=224) | Non-shortened group  (n=147) | Shortened group  (n=77) | p-value |
| --- | --- | --- | --- |
| AUC_0-24h_ ≥400 µg·h/mL | 72 (49%) | 64 (83%) | <0.001 |
| AUC_0-24h_ >500 µg·h/mL | 19 (13%) | 43 (56%) | <0.001 |
| AUC_0-24h_ >600 µg·h/mL | 1 (1%) | 17 (22%) | <0.001 |
| AUC_24-48h_ ≥400 µg·h/mL | 83 (56%) | 52 (68%) | 0.108 |
| AUC_24-48h_ >500 µg·h/mL | 39 (27%) | 27 (35%) | 0.183 |
| AUC_24-48h_ >600 µg·h/mL | 15 (10%) | 8 (10%) | 0.965 |
| Initial dose; <25 mg/kg (n=93) | Non-shortened group  (n=69) | Shortened group  (n=24) |  |
| AUC_0-24h_ ≥400 µg·h/mL | 21 (30%) | 17 (71%) | <0.001 |
| AUC_0-24h_ >500 µg·h/mL | 10 (14%) | 8 (33%) | 0.044 |
| AUC_0-24h_ >600 µg·h/mL | 1 (1%) | 3 (13%) | 0.052 |
| AUC_24-48h_ ≥400 µg·h/mL | 36 (52%) | 17 (71%) | 0.112 |
| AUC_24-48h_ >500 µg·h/mL | 18 (26%) | 7 (29%) | 0.769 |
| AUC_24-48h_ >600 µg·h/mL | 6 (9%) | 1 (4%) | 0.673 |
| Initial dose; ≥25 mg/kg (n=131) | Non-shortened group  (n=78) | Shortened group  (n=53) |  |
| AUC_0-24h_ ≥400 µg·h/mL | 51 (65%) | 47 (89%) | 0.003 |
| AUC_0-24h_ >500 µg·h/mL | 9 (12%) | 35 (66%) | <0.001 |
| AUC_0-24h_ >600 µg·h/mL | 0 (0%) | 14 (26%) | <0.001 |
| AUC_24-48h_ ≥400 µg·h/mL | 47 (60%) | 35 (66%) | 0.502 |
| AUC_24-48h_ >500 µg·h/mL | 21 (27%) | 20 (38%) | 0.190 |
| AUC_24-48h_ >600 µg·h/mL | 9 (12%) | 7 (13%) | 0.791 |
| Combined amount of the initial and second doses; <40 mg/kg (n=117) | Non-shortened group  (n=83) | Shortened group  (n=34) |  |
| AUC_0-24h_ ≥400 µg·h/mL | 33 (40%) | 27 (79%) | <0.001 |
| AUC_0-24h_ >500 µg·h/mL | 13 (16%) | 17 (50%) | <0.001 |
| AUC_0-24h_ >600 µg·h/mL | 1 (1%) | 6 (18%) | 0.003 |
| AUC_24-48h_ ≥400 µg·h/mL | 42 (51%) | 24 (71%) | 0.048 |
| AUC_24-48h_ >500 µg·h/mL | 19 (23%) | 12 (35%) | 0.168 |
| AUC_24-48h_ >600 µg·h/mL | 5 (6%) | 1 (3%) | 0.670 |
| Combined amount of the first and second doses; ≥40 mg/kg (n=107) | Non-shortened group  (n=39) | Shortened group  (n=37) |  |
| AUC_0-24h_ ≥400 µg·h/mL | 39 (61%) | 37 (86%) | 0.005 |
| AUC_0-24h_ >500 µg·h/mL | 6 (9%) | 26 (60%) | <0.001 |
| AUC_0-24h_ >600 µg·h/mL | 0 (0%) | 11 (26%) | <0.001 |
| AUC_24-48h_ ≥400 µg·h/mL | 41 (64%) | 28 (65%) | 0.911 |
| AUC_24-48h_ >500 µg·h/mL | 20 (31%) | 15 (35%) | 0.695 |
| AUC_24-48h_ >600 µg·h/mL | 10 (16%) | 7 (16%) | 0.928 |

The data are presented as number (percentage). Statistical significance was set at p <0.05. Abbreviations: AUC, area under the concentration-time curve. AUC_0-24h_, AUC on day 1; AUC_24-48h_, AUC on day 2.

**Table S2** Fine-Gray analyses of factors associated with AKI within seven days

|  | Univariate model | | | Multivariate model A | | | Multivariate model B | | |
| --- | --- | --- | --- | --- | --- | --- | --- | --- | --- |
|  | HR | (95% CI) | p-value | HR | (95% CI) | p-value | HR | (95% CI) | p-value |
| Age, per 1-year increase | 1.00 | 0.99–1.02 | 0.720 |  |  |  |  |  |  |
| Sex; female | 0.71 | 0.43–1.19 | 0.190 |  |  |  |  |  |  |
| BMI, per 1 kg/m^2^ increase | 1.02 | 0.98–1.06 | 0.380 |  |  |  |  |  |  |
| ICU | 2.23 | 1.38–3.59 | <0.001 | 1.22 | 0.64-2.33 | 0.550 | 1.41 | 0.74-2.69 | 0.290 |
| Sepsis | 1.33 | 0.77–2.29 | 0.300 |  |  |  |  |  |  |
| Septic shock | 1.89 | 0.91–3.93 | 0.088 | 0.94 | 0.42-2.14 | 0.890 | 1.03 | 0.44-2.43 | 0.940 |
| Vancomycin therapy (up to initial TDM) |  |  |  |  |  |  |  |  |  |
| Shortened interval | 1.59 | 0.94–2.39 | 0.069 | 1.27 | 0.71-2.25 | 0.420 | 1.32 | 0.73-2.38 | 0.350 |
| Loding dose, ≥25mg/kg | 1.38 | 0.81–2.34 | 0.230 |  |  |  |  |  |  |
| Vancomycin AUC |  |  |  |  |  |  |  |  |  |
| AUC_0-24h_ ≤600 µg·h/mL and AUC_24-48h_ ≤600 µg·h/mL | Reference | - | - | Reference | - | - |  |  |  |
| AUC_0-24h_ >600 µg·h/mL and AUC_24-48h_ ≤600 µg·h/mL | 1.94 | 0.50-7.53 | 0.340 | 1.77 | 0.37-8.39 | 0.470 |  |  |  |
| AUC_0-24h_ ≤600 µg·h/mL and AUC_24-48h_ >600 µg·h/mL | 3.42 | 1.72-6.80 | <0.001 | 3.68 | 1.86-7.28 | <0.001 |  |  |  |
| AUC_0-24h_ >600 µg·h/mL and AUC_24-48h_ >600 µg·h/mL | 12.17 | 5.94-24.92 | <0.001 | 13.00 | 5.96-28.35 | <0.001 |  |  |  |
| AUC_0-24h_ ≤500 µg·h/mL and AUC_24-48h_ ≤500 µg·h/mL | Reference | - | - |  |  |  | Reference | - | - |
| AUC_0-24h_ >500 µg·h/mL and AUC_24-48h_ ≤500 µg·h/mL | 1.58 | 0.47-5.29 | 0.460 |  |  |  | 1.62 | 0.44-6.00 | 0.470 |
| AUC_0-24h_ ≤500 µg·h/mL and AUC_24-48h_ >500 µg·h/mL | 2.52 | 1.20-5.28 | 0.015 |  |  |  | 3.12 | 1.44-6.80 | <0.001 |
| AUC_0-24h_ >500 µg·h/mL and AUC_24-48h_ >500 µg·h/mL | 6.01 | 3.50-10.33 | <0.001 |  |  |  | 6.89 | 3.77-12.60 | <0.001 |
| Tazobactam/Piperacillin | 3.62 | 2.25–5.83 | <0.001 | 4.38 | 2.70-7.11 | <0.001 | 5.26 | 3.18-8.70 | <0.001 |
| Catecholamine | 3.15 | 1.93–5.14 | <0.001 | 2.38 | 1.17-4.87 | 0.017 | 2.19 | 1.17-4.34 | 0.025 |
| Loop diuretics | 2.01 | 1.23–3.28 | 0.005 | 1.28 | 0.70-2.32 | 0.420 | 0.98 | 0.53-1.82 | 0.960 |

Statistical significance was set at p <0.05. To account for the increased alpha error, the Bonferroni correction was used to compare the four groups. To perform statistical analysis three times versus the control group, a p-value of <0.0167 was considered statistically significant. Abbreviations: AKI, acute kidney injury; HR, hazard ratio; CI, confidence interval; BMI, body mass index; ICU, intensive care unit; AUC, area under the concentration-time curve; AUC_24-48h_, AUC on day 2.

**Table S3** AUCs at the time of initial dose design

|  | All (n=668) | AUC_0-24h_ ≤600 µg·h/mL and AUC_24-48h_ ≤600 µg·h/mL group (n=594) | AUC_0-24h_ >600 µg·h/mL and AUC_24-48h_ ≤600 µg·h/mL group (n=14) | AUC_0-24h_ ≤600 µg·h/mL and AUC_24-48h_ >600 µg·h/mL group (n=42) | AUC_0-24h_ >600 µg·h/mL and AUC_24-48h_ >600 µg·h/mL group (n=18) | p-value |
| --- | --- | --- | --- | --- | --- | --- |
| AUC_0-24h_, µg·h/mL | 418 (343, 489) | 411 (337, 480) | 575* (493, 630) | 446 (390, 492) | 562* (444, 641) | <0.001 |
| AUC_24-48h_, µg·h/mL | 426 (353, 496) | 418 (345, 485) | 454 (378, 523) | 497* (440, 610) | 497* (390, 630) | <0.001 |

Data are presented as median (interquartile range). Each group was classified according to the AUC at the initial TDM. The Kruskal–Wallis test was used for the overall analysis, and the Dunn test was used to compare each group, with the group with neither AUC less than 600 µg·h/mL as the control group. Statistical significance was set at p <0.05.

*: Indicates a p-value of <0.0167. To account for the increased alpha error, the Bonferroni correction was used to compare the four groups. To perform statistical analysis three times versus the control group, a p-value of <0.0167 was considered statistically significant.

Abbreviations: AUC, area under the concentration-time curve. AUC_0-24h_, AUC on day 1; AUC_24-48h_, AUC on day 2.
