# Supplementary material for: Shortening the interval between the first and the second dose of vancomycin facilitates rapid achievement of the target AUC without increasing the risk of acute kidney injury, provided the AUC on the second day is appropriately controlled: a multicenter retrospective study
Source: J Pharm Health Care Sci. 2025 May 26;11:44. doi: 10.1186/s40780-025-00452-3 (PMC12105161; doi:10.1186/s40780-025-00452-3)
Supplement: Supplementary file 2 — Supplementary Material 2 [file 40780_2025_452_MOESM2_ESM.pptx]

## Slide 1
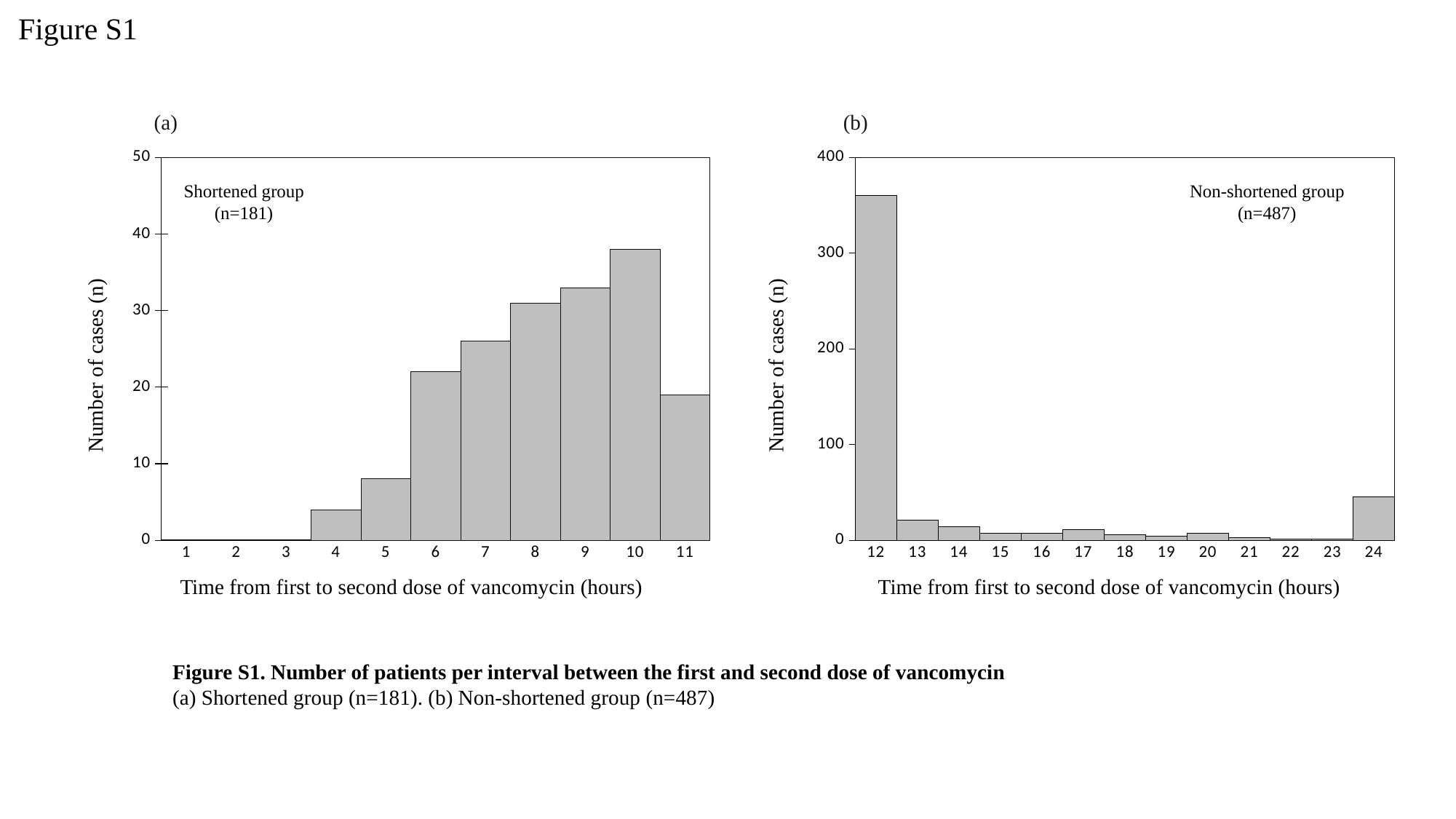

Figure S1
(a)
(b)
### Chart
| Category | |
|---|---|
### Chart
| Category | |
|---|---|
| 12 | 360.0 |
| 13 | 21.0 |
| 14 | 14.0 |
| 15 | 7.0 |
| 16 | 7.0 |
| 17 | 11.0 |
| 18 | 6.0 |
| 19 | 4.0 |
| 20 | 7.0 |
| 21 | 3.0 |
| 22 | 1.0 |
| 23 | 1.0 |
| 24 | 45.0 |Shortened group (n=181)
Non-shortened group (n=487)
Number of cases (n)
Number of cases (n)
Time from first to second dose of vancomycin (hours)
Time from first to second dose of vancomycin (hours)
Figure S1. Number of patients per interval between the first and second dose of vancomycin
(a) Shortened group (n=181). (b) Non-shortened group (n=487)

## Slide 2
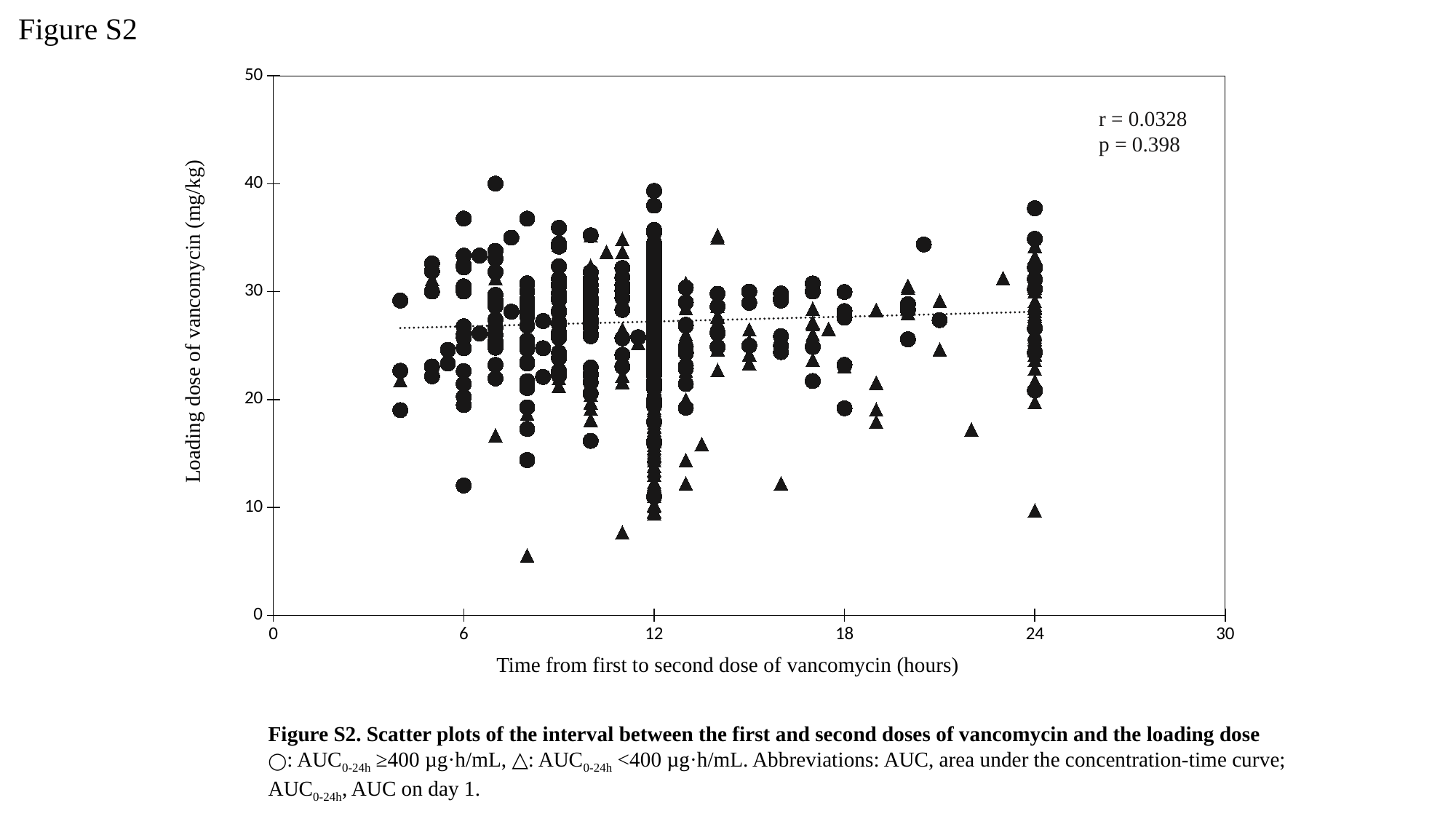

Figure S2
### Chart
| Category | 初回投与量(mg/kg) | |
|---|---|---|r = 0.0328
p = 0.398
Loading dose of vancomycin (mg/kg)
Time from first to second dose of vancomycin (hours)
Figure S2. Scatter plots of the interval between the first and second doses of vancomycin and the loading dose
◯: AUC0-24h ≥400 µg·h/mL, △: AUC0-24h <400 µg·h/mL. Abbreviations: AUC, area under the concentration-time curve; AUC0-24h, AUC on day 1.

## Slide 3
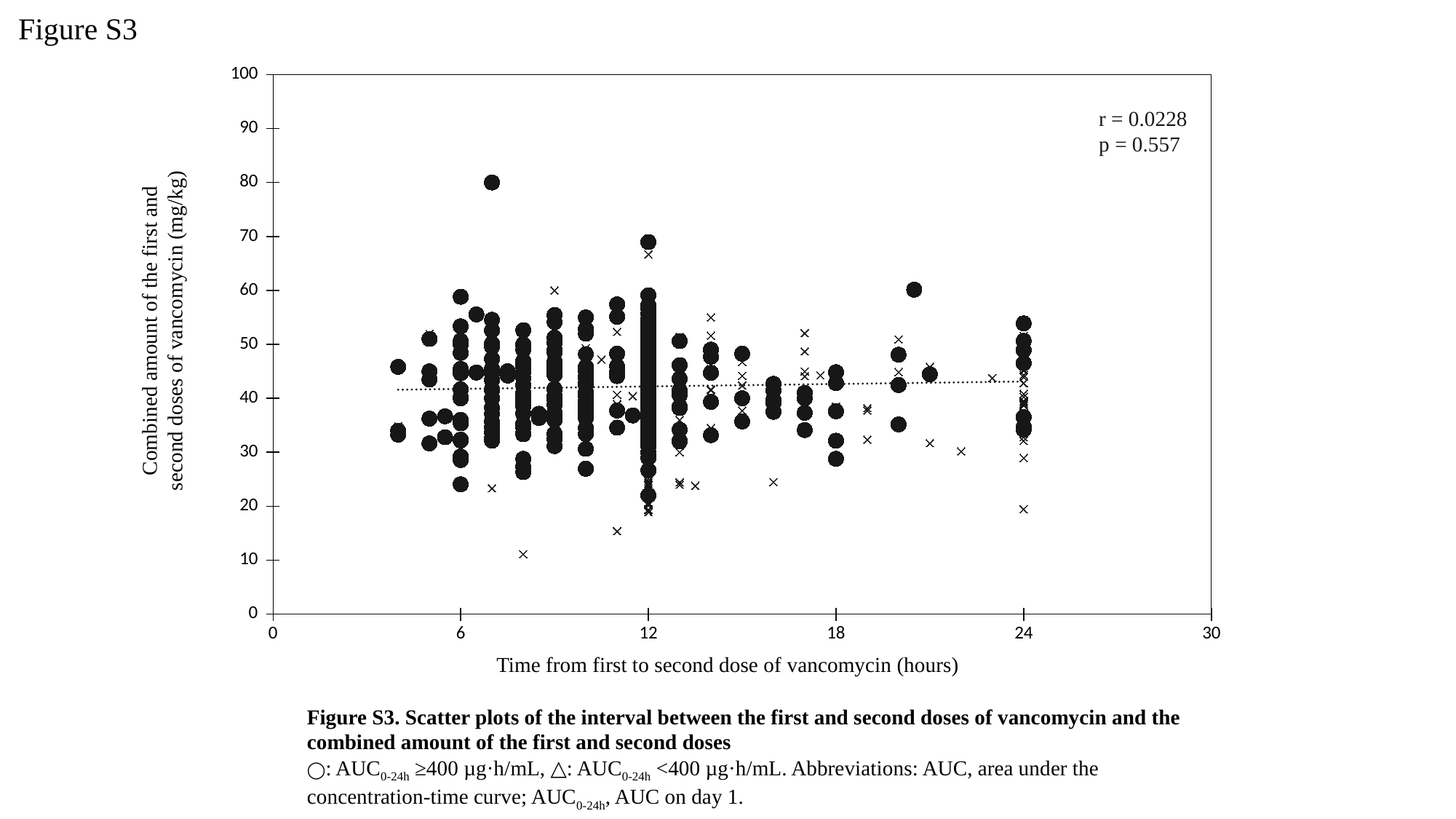

Figure S3
### Chart
| Category | 1+2回目の投与量の合計mg/kg | |
|---|---|---|r = 0.0228
p = 0.557
Combined amount of the first and second doses of vancomycin (mg/kg)
Time from first to second dose of vancomycin (hours)
Figure S3. Scatter plots of the interval between the first and second doses of vancomycin and the combined amount of the first and second doses
◯: AUC0-24h ≥400 µg·h/mL, △: AUC0-24h <400 µg·h/mL. Abbreviations: AUC, area under the concentration-time curve; AUC0-24h, AUC on day 1.

## Slide 4
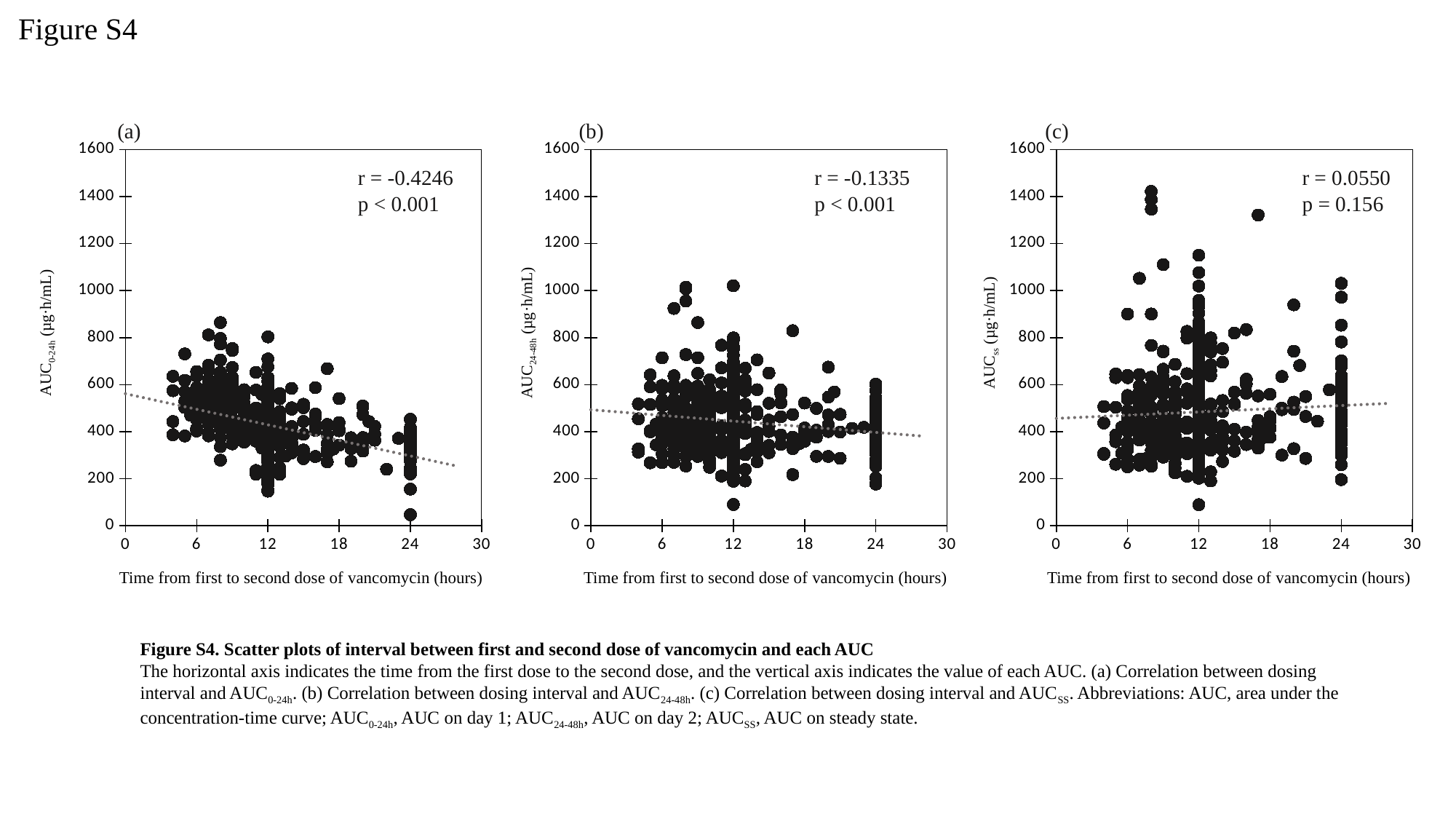

Figure S4
(a)
(b)
(c)
### Chart
| Category | 2点AUC0-24h1回目TDM(実測値,Scr調整なし) |
|---|---|
### Chart
| Category | 2点AUC24-48h1回目TDM(実測値,Scr調整なし) |
|---|---|
### Chart
| Category | 2点AUC定常1回目TDM(実測値,Scr調整なし) |
|---|---|r = -0.4246
p < 0.001
r = -0.1335
p < 0.001
r = 0.0550
p = 0.156
AUC0-24h (µg·h/mL)
AUC24-48h (µg·h/mL)
AUCss (µg·h/mL)
Time from first to second dose of vancomycin (hours)
Time from first to second dose of vancomycin (hours)
Time from first to second dose of vancomycin (hours)
Figure S4. Scatter plots of interval between first and second dose of vancomycin and each AUC
The horizontal axis indicates the time from the first dose to the second dose, and the vertical axis indicates the value of each AUC. (a) Correlation between dosing interval and AUC0-24h. (b) Correlation between dosing interval and AUC24-48h. (c) Correlation between dosing interval and AUCSS. Abbreviations: AUC, area under the concentration-time curve; AUC0-24h, AUC on day 1; AUC24-48h, AUC on day 2; AUCSS, AUC on steady state.

## Slide 5
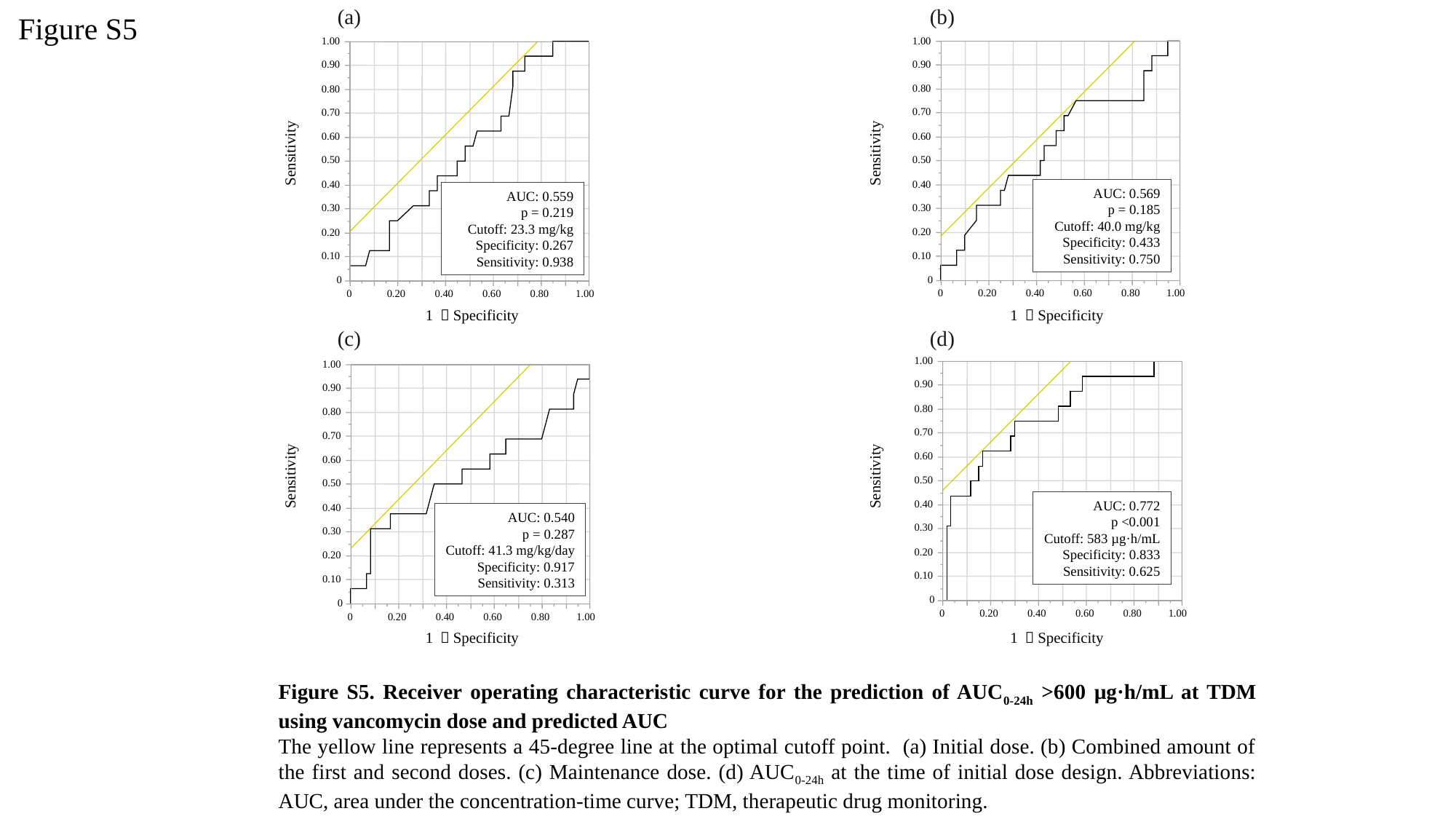

(a)
(b)
Figure S5
1.00
0.90
0.80
0.70
0.60
0.50
0.40
0.30
0.20
0.10
0
0
0.20
0.40
0.60
0.80
1.00
1.00
0.90
0.80
0.70
0.60
0.50
0.40
0.30
0.20
0.10
0
0
0.20
0.40
0.60
0.80
1.00
Sensitivity
Sensitivity
AUC: 0.569
p = 0.185
Cutoff: 40.0 mg/kg
Specificity: 0.433
Sensitivity: 0.750
AUC: 0.559
p = 0.219
Cutoff: 23.3 mg/kg
Specificity: 0.267
Sensitivity: 0.938
1 －Specificity
1 －Specificity
(c)
(d)
1.00
0.90
0.80
0.70
0.60
0.50
0.40
0.30
0.20
0.10
0
0
0.20
0.40
0.60
0.80
1.00
1.00
0.90
0.80
0.70
0.60
0.50
0.40
0.30
0.20
0.10
0
0
0.20
0.40
0.60
0.80
1.00
 AUC: 0.772
p <0.001
Cutoff: 583 µg·h/mL
 Specificity: 0.833
Sensitivity: 0.625
Sensitivity
Sensitivity
AUC: 0.540
 p = 0.287
Cutoff: 41.3 mg/kg/day
Specificity: 0.917
Sensitivity: 0.313
1 －Specificity
1 －Specificity
Figure S5. Receiver operating characteristic curve for the prediction of AUC0-24h >600 µg·h/mL at TDM using vancomycin dose and predicted AUC
The yellow line represents a 45-degree line at the optimal cutoff point. (a) Initial dose. (b) Combined amount of the first and second doses. (c) Maintenance dose. (d) AUC0-24h at the time of initial dose design. Abbreviations: AUC, area under the concentration-time curve; TDM, therapeutic drug monitoring.

## Slide 6
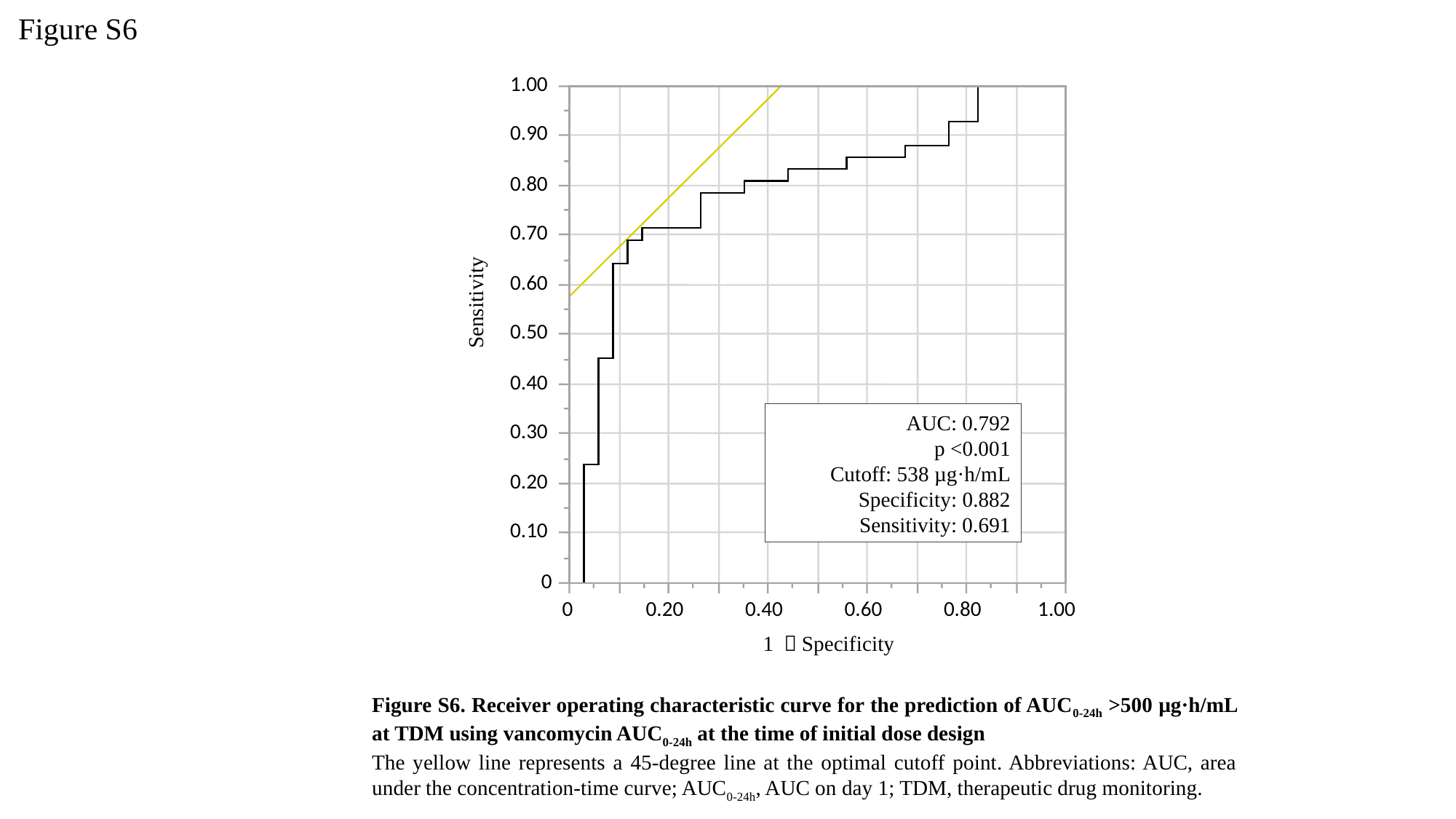

Figure S6
1.00
0.90
0.80
0.70
0.60
0.50
0.40
0.30
0.20
0.10
0
0
0.20
0.40
0.60
0.80
1.00
Sensitivity
 AUC: 0.792
p <0.001
Cutoff: 538 µg·h/mL
 Specificity: 0.882
Sensitivity: 0.691
1 －Specificity
Figure S6. Receiver operating characteristic curve for the prediction of AUC0-24h >500 µg·h/mL at TDM using vancomycin AUC0-24h at the time of initial dose design
The yellow line represents a 45-degree line at the optimal cutoff point. Abbreviations: AUC, area under the concentration-time curve; AUC0-24h, AUC on day 1; TDM, therapeutic drug monitoring.

## Slide 7
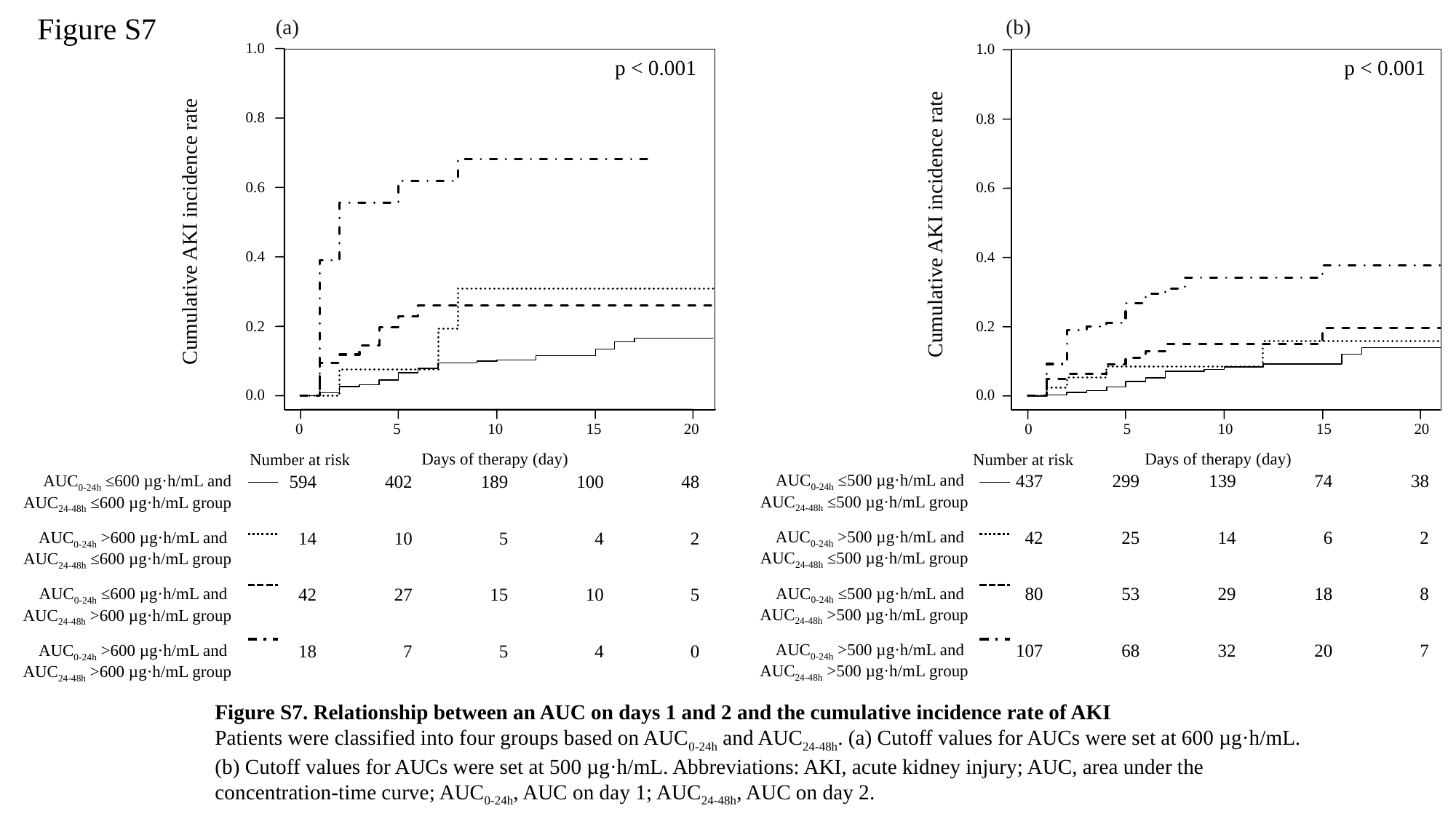

Figure S7
(a)
(b)
1.0
0.8
0.6
0.4
0.2
0.0
0
5
10
15
20
1.0
0.8
0.6
0.4
0.2
0.0
0
5
10
15
20
p < 0.001
p < 0.001
Cumulative AKI incidence rate
Cumulative AKI incidence rate
Days of therapy (day)
Days of therapy (day)
Number at risk
Number at risk
| AUC0-24h ≤500 µg·h/mL and AUC24-48h ≤500 µg·h/mL group | | 437 | 299 | 139 | 74 | 38 |
| --- | --- | --- | --- | --- | --- | --- |
| AUC0-24h >500 µg·h/mL and AUC24-48h ≤500 µg·h/mL group | | 42 | 25 | 14 | 6 | 2 |
| AUC0-24h ≤500 µg·h/mL and AUC24-48h >500 µg·h/mL group | | 80 | 53 | 29 | 18 | 8 |
| AUC0-24h >500 µg·h/mL and AUC24-48h >500 µg·h/mL group | | 107 | 68 | 32 | 20 | 7 |
| AUC0-24h ≤600 µg·h/mL and AUC24-48h ≤600 µg·h/mL group | | 594 | 402 | 189 | 100 | 48 |
| --- | --- | --- | --- | --- | --- | --- |
| AUC0-24h >600 µg·h/mL and AUC24-48h ≤600 µg·h/mL group | | 14 | 10 | 5 | 4 | 2 |
| AUC0-24h ≤600 µg·h/mL and AUC24-48h >600 µg·h/mL group | | 42 | 27 | 15 | 10 | 5 |
| AUC0-24h >600 µg·h/mL and AUC24-48h >600 µg·h/mL group | | 18 | 7 | 5 | 4 | 0 |
Figure S7. Relationship between an AUC on days 1 and 2 and the cumulative incidence rate of AKI
Patients were classified into four groups based on AUC0-24h and AUC24-48h. (a) Cutoff values for AUCs were set at 600 µg·h/mL. (b) Cutoff values for AUCs were set at 500 µg·h/mL. Abbreviations: AKI, acute kidney injury; AUC, area under the concentration-time curve; AUC0-24h, AUC on day 1; AUC24-48h, AUC on day 2.

## Slide 8
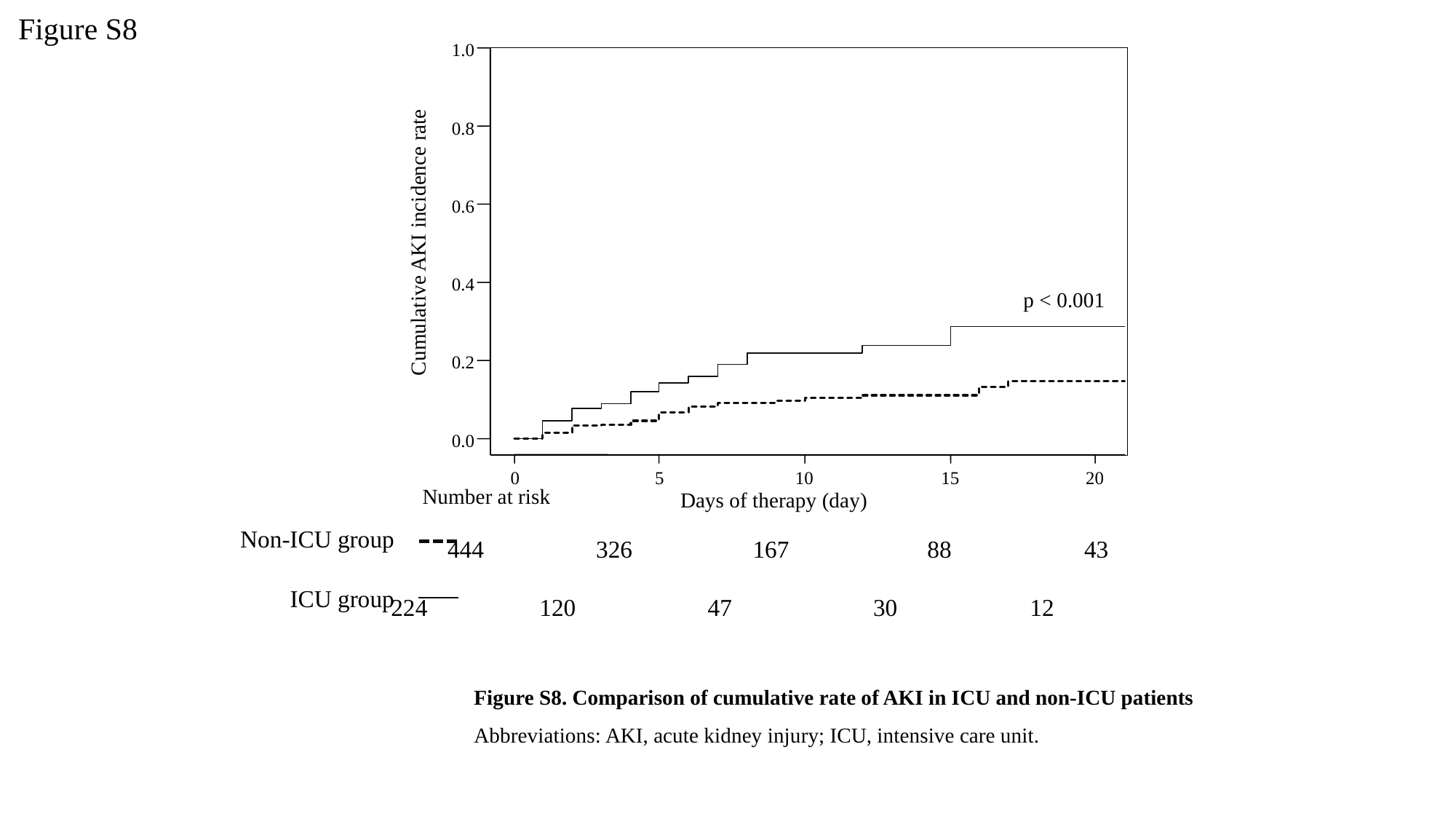

Figure S8
1.0
0.8
0.6
Cumulative AKI incidence rate
0.4
p < 0.001
0.2
0.0
0
5
10
15
20
Number at risk
Days of therapy (day)
 　　　　　 444 　 　　326 　　　167 　　　　 88　　　　　43
　　　　　　224 　 　　120 　 　　47　 　　 　30 　　　　12
Non-ICU group
ICU group
Figure S8. Comparison of cumulative rate of AKI in ICU and non-ICU patients
Abbreviations: AKI, acute kidney injury; ICU, intensive care unit.

## Slide 9
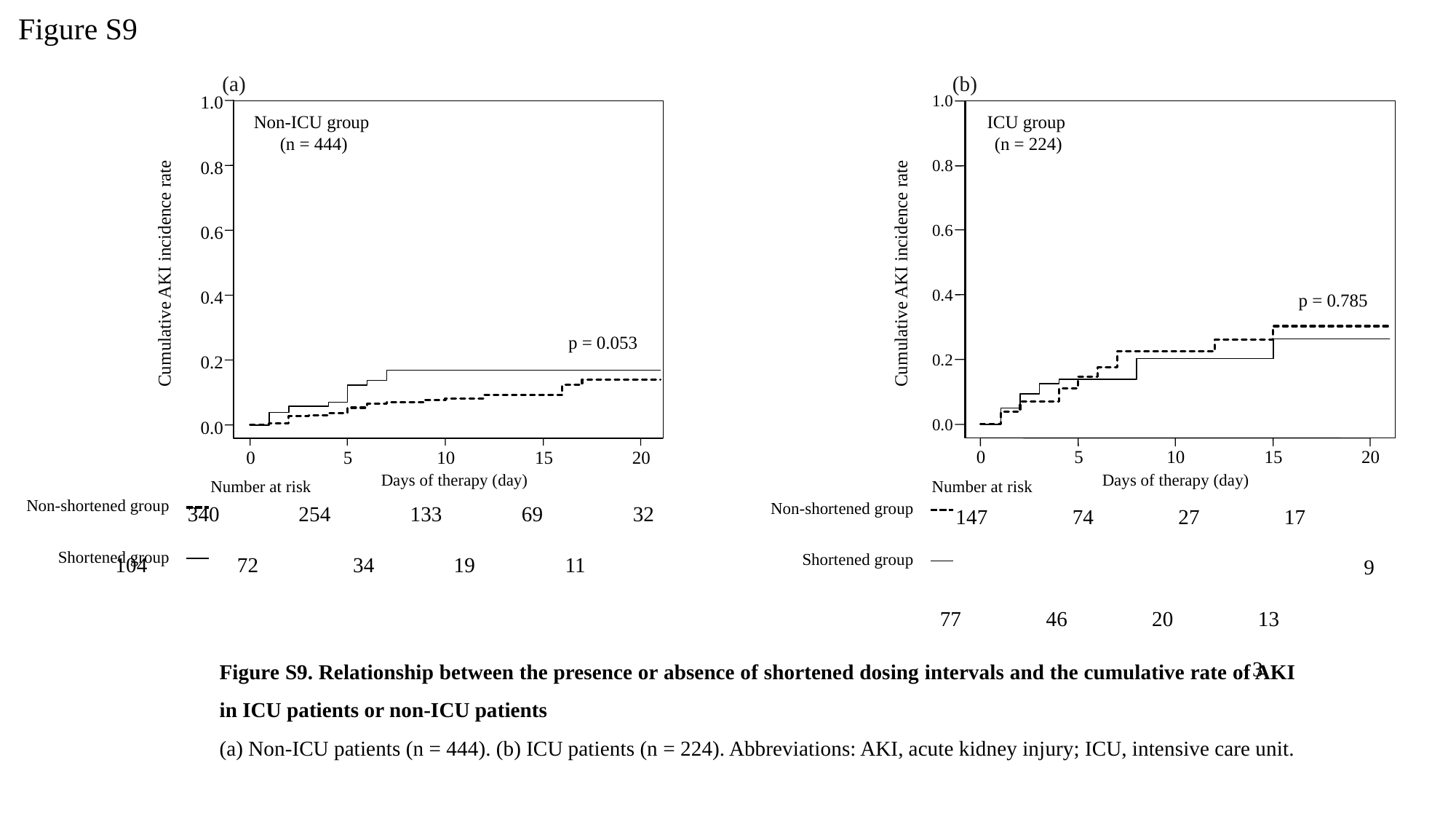

Figure S9
(a)
(b)
1.0
0.8
0.6
0.4
0.2
0.0
0
5
10
15
20
1.0
0.8
0.6
0.4
0.2
0.0
0
5
10
15
20
Non-ICU group
 (n = 444)
ICU group
 (n = 224)
Cumulative AKI incidence rate
Cumulative AKI incidence rate
p = 0.785
p = 0.053
Days of therapy (day)
Days of therapy (day)
340　 　　254 　　　133 　　　69 　　　32
104 　　72 　　　 34 　　　19 　　　11
Number at risk
Number at risk
147 　　　74 　　　27 　　　17 　　　9
77 　　　46 　　　20 　　　13 　　　3
Non-shortened group
Non-shortened group
Shortened group
Shortened group
Figure S9. Relationship between the presence or absence of shortened dosing intervals and the cumulative rate of AKI in ICU patients or non-ICU patients
(a) Non-ICU patients (n = 444). (b) ICU patients (n = 224). Abbreviations: AKI, acute kidney injury; ICU, intensive care unit.
